# Asymmetry between right and left optical coherence tomography images identified using convolutional neural networks

**DOI:** 10.1038/s41598-022-14140-x

**Published:** 2022-06-15

**Authors:** Tae Seen Kang, Woohyuk Lee, Shin Hyeong Park, Yong Seop Han

**Affiliations:** 1 Department of Ophthalmology, Gyeongsang National University Changwon Hospital, #11 Samjeongja-ro, Seongsan-gu, Changwon, 51472 Republic of Korea; 2 Department of Ophthalmology, Gyeongsang National University College of Medicine, Institute of Health Sciences, Jinju, Republic of Korea

**Keywords:** Eye diseases, Computer science

## Abstract

In a previous study, we identified biocular asymmetries in fundus photographs, and macula was discriminative area to distinguish left and right fundus images with > 99.9% accuracy. The purposes of this study were to investigate whether optical coherence tomography (OCT) images of the left and right eyes could be discriminated by convolutional neural networks (CNNs) and to support the previous result. We used a total of 129,546 OCT images. CNNs identified right and left horizontal images with high accuracy (99.50%). Even after flipping the left images, all of the CNNs were capable of discriminating them (DenseNet121: 90.33%, ResNet50: 88.20%, VGG19: 92.68%). The classification accuracy results were similar for the right and left flipped images (90.24% vs. 90.33%, respectively; *p* = 0.756). The CNNs also differentiated right and left vertical images (86.57%). In all cases, the discriminatory ability of the CNNs yielded a significant *p* value (< 0.001). However, the CNNs could not well-discriminate right horizontal images (50.82%, *p* = 0.548). There was a significant difference in identification accuracy between right and left horizontal and vertical OCT images and between flipped and non-flipped images. As this could result in bias in machine learning, care should be taken when flipping images.

## Introduction

Optical coherence tomography (OCT) is an imaging modality providing high-resolution cross-sectional and three-dimensional images of living tissue^1^. OCT can be used to quickly and safely examine eyes at the cellular level. OCT has been widely used for diagnosing retinal and optic disc diseases, is readily accessible for ophthalmologists, and is being used increasingly in dermatology^2^ and cardiology^2^.

A convolutional neural network (CNN) is an image analysis method that has developed rapidly in recent years. The multi-layered structure of the visual cortex inspired the development of CNNs^3^. CNNs show high ability to analyze and classify images. In recent studies, the classification ability of some CNNs was similar to that of physicians^4,5^.

The accuracy of CNNs for diagnosing ophthalmic diseases has been evaluated in numerous studies^6^, including ones on retinal disease^7–11^ and glaucoma^12^, in which CNNs were able to determine patient’ age, sex, and even smoking status from retinal images. A fundus image of the left eye appears as a mirror image of the fundus image of the right eye. In a previous study of CNNs, we identified asymmetries in fundus photographs^13^. However, fundus images can also be affected by several factors such as the camera lens, flash, and room lighting conditions. As OCT is free from these factors, we try to evaluate the asymmetry of right and left eyes using high-resolution OCT with CNN models.

## Results

### Baseline characteristics of image sets

Medical charts of patients who visited Gyeongsang National University Changwon Hospital from February 2016 to December 2020 were reviewed retrospectively. A total of 3,238,650 macular images were taken from 9274 patients between 2016 and May 2021. We selected 129,546 median images from among the total of 3,238,650 images. There were 33,366 right horizontal, 31,211 right vertical, 33,429 left horizontal, and 31,540 left vertical OCT images.

We split the images in each set into training, validation, and testing sets according to a 8:1:1 ratio. Sets 1–5 consisted of horizontal images (33,366 right horizontal and 33,429 left horizontal images). Of these 66,795 images, 6680 were used for model testing. During CNN learning, 53,435 images were used to train the models and 6680 for model validation. Sets 6 and 7 consisted of vertical images (31,211 right vertical and 31,540 left vertical images). Of the 62,751 images, 6276 were used for testing. During CNN learning, 50,199 images were used for training and 6276 for validation. Set 8 consisted of only 33,366 right horizontal images, split into 3337 images for testing, 26,692 for training, and 3337 for validation.

### Comparison of right and left horizontal OCT images (Set 1; R^h^L^h^D^121^)

**Figure 1:**
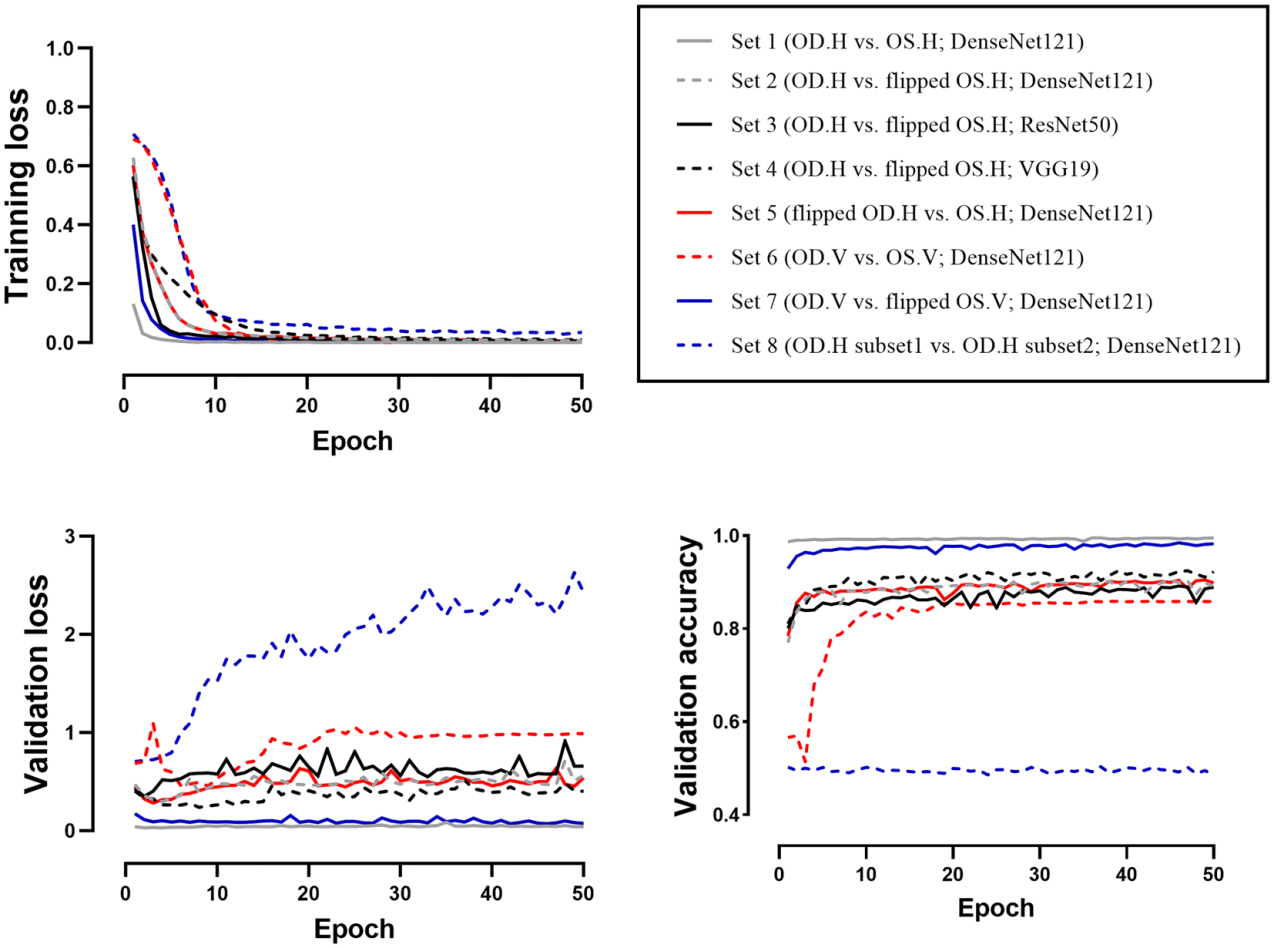
Training and validation results. The training loss of Sets 1–7 approached zero. In Set 1, images of right and left eyes were easily distinguished, with the validation loss approaching zero and the validation accuracy thus approaching 1.0. In Sets 2–5, similar validation loss (~ 0.4) and validation accuracy (~ 90%) were obtained. For Set 6, which consisted of vertical images, the accuracy was slightly inferior compared to Sets 2–5. Set 7 showed the second-highest validation accuracy. In contrast to Sets 1–7, overfitting was observed in Set 8; in this set, training loss converged whereas validation loss diverged. The validation accuracy of Set 8 was around 0.5 and failed to improve over the learning period. *OD* oculus dexter, *OS* oculus sinister, *H* horizontal, *V* vertical.

**Figure 2:**
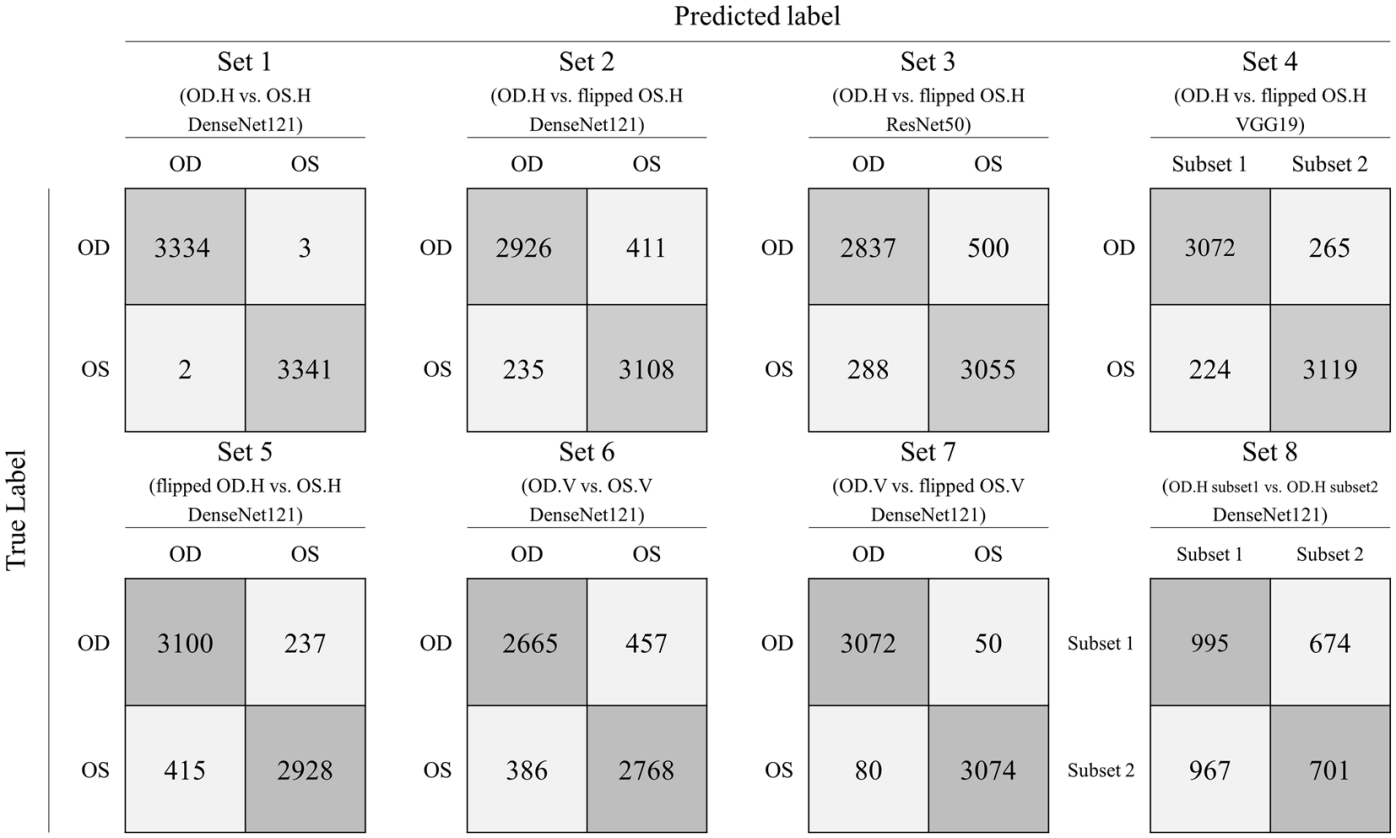
Confusion matrix for each test set. The classification accuracy for Set 1 was 99.93%, which was the highest among all sets. (AUC = 0.999, *p* < 0.001). Sets 2–5 showed test accuracies of ~ 90% (90.33%, 88.20%, 92.68%, and 90.24%, respectively; AUC: 0.902, 0.882, 0.927, and 0.902, respectively; all *p* values < 0.001). Set 6 showed a test accuracy of 86.57% (AUC = 0.866, *p* < 0.001). The accuracy of Set 7 was 97.93%, which was the second-highest (AUC = 0.979, *p* < 0.001). The results for Sets 1–7 were statistically significant. Set 8 showed 50.82% classification accuracy (AUC = 0.505, *p* = 0.548). *OD* oculus dexter, *OS* oculus sinister, *H* horizontal, *V* vertical.

We classified the right and left horizontal OCT images using the CNN model. After the 50th epoch, the validation accuracy was 99.50% (Fig. 1). Of the 6680 test set images, 6675 were correctly labeled by the CNNs, for a test accuracy of 99.93% (AUC = 0.999, *p* < 0.001, Fig. 2).

### Comparison of right and flipped left horizontal OCT images (Sets 2–4; R^h^_f_L^h^)

We classified the non-flipped right horizontal OCT images and flipped left horizontal images using DenseNet121, ResNet50, and VGG19. The numbers of images in Sets 2–4(R^h^_f_L^h^) were the same as in Set 1(R^h^L^h^D^121^). After the 50th epoch, the validation accuracy was 92.97%, 88.92%, and 92.23% in Set 2(R^h^_f_L^h^D^121^), Set 3(R^h^_f_L^h^R^50^), and Set 4(R^h^_f_L^h^V^19^), respectively (Fig. 1). The test accuracies were around 90% (90.33%, 88.20%, and 92.68%, respectively; AUC: 0.902, 0.882, and 0.927, respectively; all *p* values < 0.001, Fig. 2). The AUCs differed significantly in the ROC curve comparisons (all *p* values < 0.001).

### Comparison of flipped right and non-flipped left horizontal OCT images (Set 5; _f_R^h^L^h^D^121^)

Set 5(_f_R^h^L^h^D^121^) comprised horizontally inverted versions of the images in Set 2(R^h^_f_L^h^D^121^). As we flipped only the left horizontal images in other Sets, it could cause bias. We tried to verify the results by flipping the right eye images. The DenseNet121 model classified the flipped right horizontal images and non-flipped left horizontal images. After the 50^th^ epoch, the validation accuracy was 89.83% (Fig. 1). The test accuracy was 90.24% (AUC: 0.902, *p* < 0.001, Fig. 2). In comparison to the ROC curve analysis for Set 2(R^h^_f_L^h^D^121^), the AUCs were not significantly different (_f_R^h^L^h^D^121^ vs. R^h^_f_L^h^D^121^; *p* = 0.756).

### Comparison of right and left vertical OCT images (Set 6; R^v^L^v^D^121^)

We classified the right and left vertical untransformed images using the DensdNet121 model. After the 50th epoch, the validation accuracy was 85.86% (Fig. 1). Of the 6276 test set images, 5433 were correctly labeled by the CNNs, for a test accuracy of 86.57% (AUC = 0.866, *p* < 0.001, Fig. 2). The AUC was significantly lower than that of Set 1(R^h^L^h^D^121^; AUC = 0.999) and Set 2(R^h^_f_L^h^D^121^; AUC = 0.902) (all *p* values < 0.001).

### Comparison of right and flipped left vertical OCT images (Set 7; R^v^_f_L^v^D^121^)

We classified the right and flipped left vertical untransformed images using the DensdNet121 model. After the 50th epoch, the validation accuracy was 98.26% (Fig. 1). Of the 6276 test set images, 6146 were correctly labeled by the CNNs, for a test accuracy of 97.93% (AUC = 0.979, *p* < 0.001, Fig. 2). The AUC was significantly higher than that of Set 6 (R^v^L^v^D^121^ vs. R^v^_f_L^v^D^121^; *p* < 0.001).

### Comparison between randomly distributed right horizontal OCT images (Set 8; R^h^R^h^D^121^)

Set 8(R^h^R^h^D^121^) was designed to test overfitting, with DenseNet121 applied to classify randomly selected right horizontal images. Although the training accuracy increased and training loss decreased, the validation accuracy after the 50th epoch was only 48.55%. Of the 3337 test set images, 1696 (50.82%) were classified correctly (AUC = 0.505, *p* = 0.548, Fig. 2). Unlike Sets 1–7, Set 8 displayed an irregular CAM pattern that emphasized the outside regions of the vitreous and sclera.

## Discussion

OCT is a novel imaging modality that provides high-resolution cross-sectional images of the internal microstructure of living tissue^1^. The low-coherence light of OCT penetrates the human retina and is then reflected back to the interferometer to yield a cross-sectional retinal image^14^. Retinal OCT images consist of repeated hyporeflective and hyperreflective layers. Hyperreflective layers in OCT include the retinal nerve fiber layer, inner plexiform layer, outer plexiform layer, external limiting membrane, ellipsoid zone, and retinal pigmented epithelium. Hyporeflective layers include the ganglion cell, inner nuclear, and outer nuclear layers. The choroid and parts of the sclera also appear in OCT images^15^.

Our previous study^13^ showed that left fundus images are not mirror-symmetric with respect to right fundus images. CNNs are capable of distinguishing the left from right fundus with an accuracy greater than 99.9%. However, it is important to consider the various factors that can affect fundus photography outcomes. In fundus photography, light from the flashlight reflects off the retina and enters the sensor of the fundus camera; a sensor then examines the wavelength and strength of the light. According to the working principle of a fundus camera, fundus images may be affected by the type and location of the light source and sensor, as well as by reflection and various environmental factors^16^. However, OCT is a completely different modality free from these confounding factors; high-quality cross-sectional OCT images allow visualization of the anatomy.

Our CNNs showed 99.93% classification accuracy for bilateral horizontal OCT images (Set 1; R^h^L^h^D^121^). This result is not surprising because the thick retinal nerve fiber layer (RNFL), which consists of the papillomacular bundle, is on the nasal side of the fovea but not the temporal side; in addition, large blood vessels are concentrated on the nasal side. Notably, CAM highlighted not only the RNFL but also the entire thickness of the parafoveal retina. These results indicate that CNNs are capable of recognizing anatomical asymmetry based on the anatomical information of every layer of the retina, choroid, and sclera, as well as the RNFL.

The human eye cannot distinguish a right horizontal OCT from a flipped left horizontal OCT, as the images largely coincide with each other. To examine this problem, we used image sets (Sets 2–4; R^h^_f_L^h^) to train DenseNet121, ResNet50, and VGG19. Although the classification accuracy differed among the models, all of the CNNs showed around 90% accuracy to distinguish for left and right horizontal OCT images. Thus, CNNs can discriminate horizontal OCT images that are not mirror-symmetric.

However, we could not fully explain the CAM results of Sets 2–4(R^h^_f_L^h^). CAM was used to analyze the last layers of the CNN, and the results may have been affected by the model structure. The classification results for DenseNet121 were similar between Set 1(R^h^L^h^D^121^) and Set 2(R^h^_f_L^h^D^121^). The ResNet50 CAM for Set 3(R^h^_f_L^h^R^50^) displayed a vertically linear pattern that included the vitreous region and the region outside the sclera. Given that the region surrounding the sclera is irrelevant for OCT images, this indicates an error. The AUC for Set 3(R^h^_f_L^h^R^50^) was significantly lower than those of Set 2(R^h^_f_L^h^D^121^) and Set 4(R^h^_f_L^h^V^19^); this was attributable to uninterpretable CAM results. The VGG19 CAM for Set 4(R^h^_f_L^h^V^19^) highlighted the outer retina parafoveal and foveolar regions, different from Set 2(R^h^_f_L^h^D^121^) and Set 3(R^h^_f_L^h^R^50^).

As we flipped only the left horizontal images in other Sets, it could induce bias. The purpose of Set 5(_f_R^h^L^h^D^121^) was to verify whether the flip function in the NumPy package has errors. Set 5(_f_R^h^L^h^D^121^) consisted of horizontally inverted versions of the images in Set 2(R^h^_f_L^h^D^121^). If image flipping did not distort the images, we would expect to obtain similar results between Set 2(R^h^_f_L^h^D^121^) and Set 5(_f_R^h^L^h^D^121^). Classification accuracy was similar between the two sets (R^h^_f_L^h^D^121^, 90.33%; _f_R^h^L^h^D^121^, 90.24%), and the AUCs were not significantly different (*p* = 0.756). Through this, we found that there was no difference between flipping the left eye images and the right eye images.

Set 6(R^v^L^v^D^121^) consisted of vertical images of the right and left eyes. It is believed that vertical images of the two eyes are symmetrical; thus, we did not expect the CNNs to distinguish them. Set 6(R^v^L^v^D^121^) images were unmodified, similar to Set 1(R^h^L^h^D^121^). The CNNs distinguished the right and left vertical OCT images with relatively high accuracy (86.57%, AUC = 0.866, *p* < 0.001). However, the accuracy for Set 6 images was significantly lower than for Sets 1 and 2. The CAM result for Set 6 were also different from Set 1(R^h^L^h^D^121^) and Set 2(R^h^_f_L^h^D^121^). For Set 6(R^v^L^v^D^121^) images, CAM highlighted not only the parafovea, but also the fovea. In a previous study using fundus photography^13^, CAM brightly highlighted the temporal parafovea and moderately highlighted the fovea. It is possible that the asymmetric differ on the location of the retina, and the temporal parafovea may have a larger asymmetric than the superior and inferior parafovea. This could be explained by asymmetry differing according to retina location; additional research is required to test this hypothesis.

Set 7(R^v^_f_L^v^D^121^) comprised OCT images of the upper and lower halves of the eye. Several studies^17–19^ have demonstrated macular and choroidal asymmetry between the upper and lower halves of the eyes. In this study, the classification accuracy was second-highest for Set 7(R^v^_f_L^v^D^121^), supporting previous studies. CAM highlighted the thickness of the parafoveal retina and choroid. The AUC of Set 7(R^v^_f_L^v^D^121^) was significantly higher compared to that of Set 6(R^v^L^v^D^121^), which also supports previous studies showing that the upper and lower halves of the retina and choroid are not identical.

Set 8(R^h^R^h^D^121^) was designed to test overfitting, which is a common problem with CNNs. The results for Sets 1–7 may have resulted from overfitting, in which a CNN would show similar results for any random OCT image. The reliability of our results would be demonstrated by an inability of the CNNs to distinguish among uniform images. The CNNs could not accurately discriminate Set 8(R^h^R^h^D^121^) images (*p* = 0.548), although training loss decreased. This result indicates that the classification results for Sets 1–7 were not affected by overfitting.

We observed asymmetry between the left- and right-eye OCT images. Cameron et al.^20^ also observed asymmetry; however, they were unable to identify specific asymmetric components. Wagner et al.^21^ reported that the “angles between the maxima of peripheral RNFL thickness” were higher in right than left eyes, and that RNFL asymmetry could be influenced by the locations of the superotemporal retinal artery and vein^22^. The retinal vascular system also exhibits interocular asymmetry. Leung et al^23^ reported that the mean central retinal arteriolar equivalent of right eyes was 3.14 µm larger than that of left eyes. In this study, the CNNs were well-capable of recognizing asymmetry.

Based on our results and previous studies using fundus imagery^24–26^, it seems clear that CNNs can distinguish several features through analyzing retinal images that cannot be resolved by humans; that is, CNNs can determine patient age, sex, and smoking status. Our CNNs identified several features distinguishing left- and right-eye images that cannot be detected by humans. The results were similar after resetting the CNNs many times. Therefore, we assume that there are hidden patterns in gray-scale OCT images detectable only by CNNs. One possible hypothesis is that the human brain has limited multi-tasking capacity compared to the computer. Human cognition has limitations in processing multiple inputs at the same time^27^. For example, in the “Where’s Wally?” visual search task^28^, the human brain has difficulty processing the seven salient features (a red-striped long-sleeved T-shirt, jeans, round glasses, a hat, a chin, and curly hair) simultaneously, whereas a computer can do this easily^29^. The numbers of filters in the last layer of DenseNet121, ResNet50, and VGG19 are 1024, 2048, and 512, respectively. In theory, each filter can find a different feature. Thus, DenseNet121 can process 1024 features, which is beyond human capabilities.

The main strength of this study was that we included images of both normal and pathological eyes. It seems that there are significant biocular asymmetries in both healthy and pathological eyes. It would be interesting to analyze asymmetry according to disease type and progression, which could aid the development of a scale for measuring normal structure degradation/destruction. In addition, we used “lossless” BMP and unmodified images (except for the cropping and flipping processes). However, only one OCT device (Spectralis SD-OCT; Heidelberg Engineering) was used, and all analyses were conducted at a single institution. Thus, future studies should compare multiple OCT systems.

In conclusion, we hypothesized that OCT images of the right and left eyes are mirror-symmetric. However, we found asymmetry in both vertical and horizontal OCT images of the right and left eyes. To our knowledge, this is the first machine learning study to assess differences in OCT images of the left and right eyes. Our CNNs could accurately distinguish left- and right-eye OCT images. However, asymmetry may introduce bias into CNN results; thus, care should be taken when flipping images during preprocessing, given the possible impact of bias on evaluations of diseases that involve the macula, such as age-related macular degeneration and diabetic macular edema.

## Methods

### Study design

This retrospective study was approved by the Institutional Review Board of Gyeongsang National University Changwon Hospital (GNUCH 2020-07-009). The procedures used in this study followed the principles of the Declaration of Helsinki. The requirement for informed patient consent was waived by the Institutional Review Board of Gyeongsang National University Changwon Hospital due to the retrospective nature of the study.

### Image acquisition protocol

**Figure 3:**
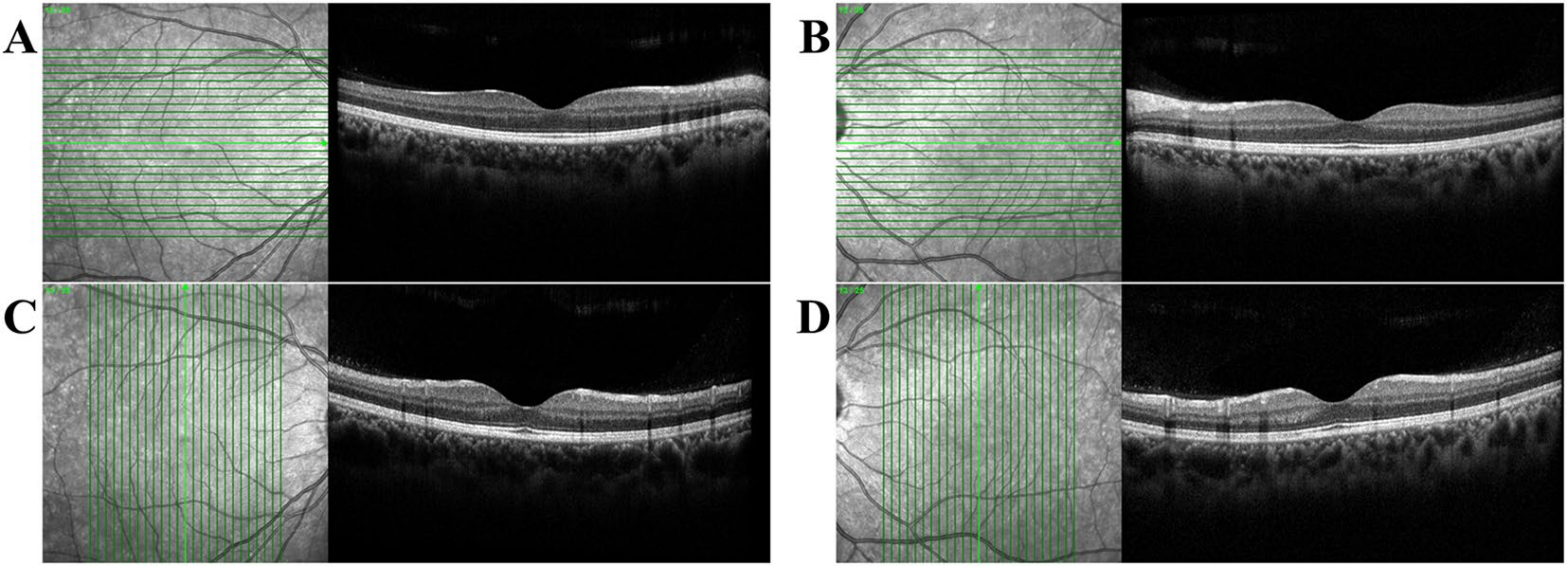
Optical coherence tomography (OCT) images used in the study. Twenty-five OCT images were obtained during one examination; only the median (13th) images were analyzed. **A** Right horizontal OCT image, **B** Left horizontal OCT image, **C** Right vertical OCT image, **D** Left vertical OCT image. In horizontal images (**A**,**B**), the optic disc is positioned on the right side of the right eye (**A**) and left side of the left eye (**B**). Since the retinal nerve fiber layer (RNFL) and major retinal vessels gather from the optic disc, the RNFL is thicker on the optic disc than on the opposite side. Also, shadows of the retinal vessels are easily identifiable on the optic disc side. As the optic discs of both eyes are located on the opposite sides, the horizontal images of the right and left eyes look like mirror images. In vertical images (**C**,**D**), the left side is the inferior part of the fovea (bottom of vertical green line), and the right side is the superior part of the fovea (top of vertical green line). Because the inferior and superior parts are located at similar distances from the optic disc, the RNFL thickness and the vascular shade density are similar. The vertical images of the right eye (**C**) and left eye (**D**) are difficult to discriminate.

An expert examiner evaluated the retinas with a Spectralis SD-OCT device (Heidelberg Engineering, Heidelberg, Germany). The system acquired 40 k A-scans per second, with an axial resolution of 3.9 μm/pixel and transverse resolution of 5.7 μm/pixel. Twenty-five cross-sectional images were taken with an interval of 240 μm. Each cross-sectional image consisted of 768 A-scans and subtended an angle of 30° (Fig. 3). The examiner took a horizontal cross-sectional image of the macula, followed by vertical cross-sectional images. We accessed these images using automated programs written in AutoIt and saved them in the bitmap (BMP) format. Only the 13th image (i.e., the median image of 25 consecutive OCT images) was analyzed. We included all cases in the analysis to reduce selection bias. The cases included various retinal diseases such as epiretinal membrane, macular hole, and rhegmatogenous retinal detachment; some eyes were filled with gas, air, or silicone oil, etc.

### Data pre-processing

**Figure 4:**
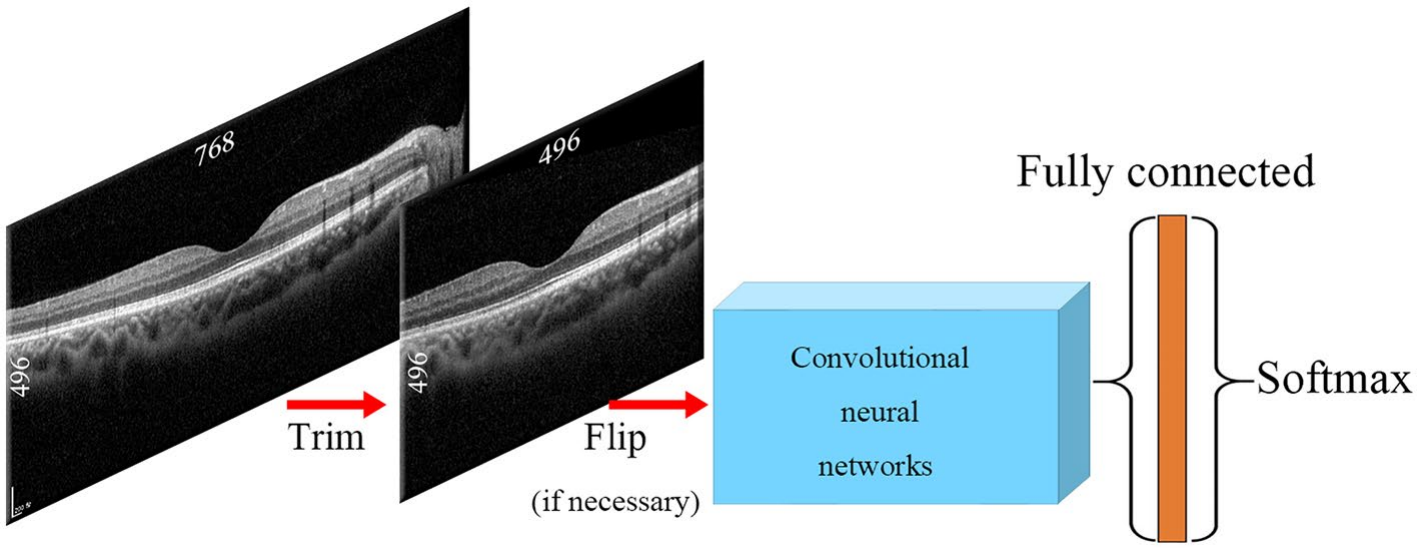
Diagram of the image processing procedure. The images were trimmed from 768 × 496 to 496 × 496 pixels. If needed, the images were flipped horizontally. The processed images were loaded into convolutional neural networks (CNNs) embedded in the Keras package and attached to the fully connected and Softmax layers.

We did not perform data augmentation. We trimmed and flipped the images using the Python packages OpenCV and NumPy^30^. The size of the original OCT images was 768 × 496 pixels. We trimmed 136 pixels at both ends of each image to remove boundary artifacts, leaving only the macula (496 × 496 pixel-size image, Fig. 4). The trimmed 496 × 496 pixel image corresponds to about 19.4° of macular.

### CNN model

Transfer learning is a method for generating a new model using a previously trained model and weights. We used a pre-trained model to improve accuracy^31–34^, as previous studies have demonstrated that the features of pre-trained models are transferable to ophthalmology data^33^. Densenet121^35^, ResNet50^36^, and VGG19^37^, embedded in the Keras package, showed excellent ability to distinguish left and right fundus photographs^13^. The outputs of DenseNet121, ResNet50, and VGG19 were connected to the fully connected layer and Softmax layer. We used categorical cross-entropy loss functions and Adam as the gradient descent optimization algorithm.

### Image sets

**Figure 5:**
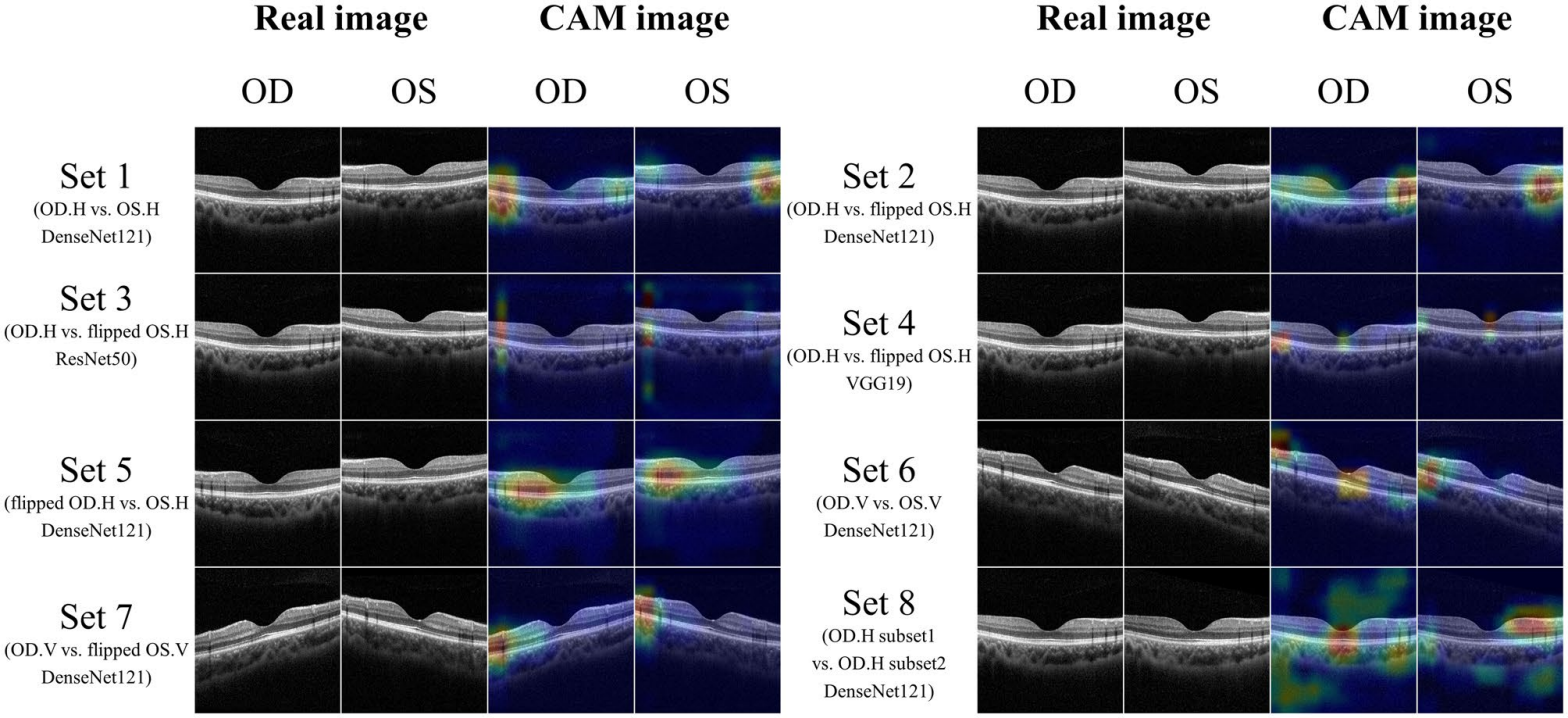
Image sets and their corresponding class activation mapping (CAM) results. Set 1 consisted of horizontal images of right and left eyes. Papillomacular retinal nerve fiber layers with high reflectivity were shown on the nasal side of the image. CAM highlights both ends of parafoveal regions of images. Sets 2–4 consisted of right horizontal images and flipped left horizontal images. Papillomacular retinal nerve fiber layers with high reflectivity were shown in the right-side of OCT images. CAM patterns in Sets 2–4 differed: DenseNet121 highlighted the parafovea, whereas ResNet50 highlighted only the parafovea and VGG19 both the parafovea and macula. Set 5 consisted of flipped right and left horizontal images; CAM highlighted the parafovea, similar to Set 2. Set 6 consisted of vertical OCT images of right and left eyes. From Set 6, heatmaps highlighting the parafovea and macular region were produced. Set 7 comprised non-flipped right vertical images and flipped left vertical OCT images; the ends of the parafoveal regions were highlighted. Set 8 consisted only of right horizontal images. Unlike Sets 1–7, Set 8 images showed an irregular pattern, emphasizing the vitreous region and outside of the sclera. *OD* oculus dexter, *OS* oculus sinister, *H* horizontal, *V* vertical.

Eight image sets were prepared (Fig. 5). Set 1(R^h^L^h^D^121^) consisted of horizontal OCT images of right and left eyes without any transformation. Sets 2–4(R^h^_f_L^h^) comprised non-flipped right horizontal images and horizontally flipped left horizontal images. We tested different CNNs on the same image dataset to check for asymmetry. Set 5(_f_R^h^L^h^D^121^), which consisted of flipped right horizontal images and non-flipped left horizontal images, showed an inversed dataset to Sets 2–4. Set 6(R^v^L^v^D^121^) consisted of vertical images of right and left eyes without any transformation. Set 7(R^v^_f_L^v^D^121^) consisted of non-flipped right vertical images and horizontally flipped left vertical images. The images in Sets 1–7 were of right and left eyes. The purpose of Set 8(R^h^R^h^D^121^) was to verify the overfitting problem that often occurs in CNNs. Set 8(R^h^R^h^D^121^) consisted of only non-flipped right horizontal images. The images were randomly divided into subsets 1 and 2.

### Class activation mapping

We used class activation mapping (CAM)^38^ to better understand how the CNNs worked. CAM uses heatmaps to identify the areas used by CNNs to make decisions. Hotter areas carry more weight in CAM heatmaps and are more important in the CNN class discrimination process. Using CAM, we identified locations that carried more weight in the final convolutional and classification layers.

### Software

Python (version 3.7.9) was used for this study. The CNN model consisted of TensorFlow 2.4.1, Keras 2.4.3, OpenCV 4.5.1.48, and NumPy 1.19.5. The performance of each CNN model was evaluated by calculating the accuracy of the test set. The central processing unit used to train the CNN model was an Intel® Core™ i9-10980XE system (Intel Corp., Santa Clara, CA, USA), equipped with a GeForce RTX 3090 graphics card (Nvidia Corp., Santa Clara, CA, USA). We analyzed the results of the test set using SPSS for Windows statistical software (version 24.0; SPSS Inc., Chicago, IL, USA). We drew receiver operating characteristic (ROC) curves with test set results and computed the area under the ROC curve (AUC). The *p* values for the ROC curves were calculated with SPSS software. We compared AUC of two ROC curves using MedCalc Statistical Software for Windows (version 19.2.6; MedCalc, Ostend, Belgium). Statistical significance was set at a *p* value of < 0.05.

## Data Availability

Data supporting the findings of the current study are available from the corresponding author upon reasonable request.
